# Activation of Sirtuin 1 Attenuates High Glucose-Induced Neuronal Apoptosis by Deacetylating p53

**DOI:** 10.3389/fendo.2018.00274

**Published:** 2018-05-28

**Authors:** Xiajie Shi, Linhua Pi, Shanlei Zhou, Xin Li, Fangyuan Min, Shan Wang, Zhenqi Liu, Jing Wu

**Affiliations:** ^1^Department of Endocrinology, Xiangya Hospital, Central South University, Changsha, China; ^2^Department of Metabolism and Endocrinology, The Second Xiangya Hospital, Central South University, Changsha, China; ^3^Department of Pharmaceutical Engineering, College of Chemistry and Chemical Engineering, Central South University, Changsha, China; ^4^Division of Endocrinology and Metabolism, Department of Medicine, University of Virginia Health System, Charlottesville, VA, United States

**Keywords:** diabetes, cognitive impairment, sirtuin 1, neuronal apoptosis, p53

## Abstract

Diabetes mellitus (DM) has been proven to be a key risk factor for cognitive impairment. Previous studies have implicated hippocampal neuronal apoptosis in diabetes-related cognitive impairment. However, the underlying mechanism remains unknown. Sirtuin 1 (SIRT1) is a protein deacetylase depended on nicotinamide adenine dinucleotide. Furthermore, it is indispensable in normal learning and memory. Whether SIRT1 is taken part in diabetes-induced neuronal apoptosis and thus involve in the development of diabetic cognitive impairment is still not clear. To address this issue, we examined the possible role of SIRT1 in hippocampal neuronal apoptosis in streptozotocin**-**induced diabetic mice. Furthermore, the possible mechanism was investigated in high glucose-induced SH-SY5Y cells. We found that downregulation of the activity and expression of SIRT1 was associated with increased hippocampal neuronal apoptosis in mice. *In vitro*, cell apoptosis induced by high glucose which was accompanied by a downregulation of SIRT1 and an increased acetylation of p53. On the contrary, activation of SIRT1 using its agonist resveratrol ameliorated cell apoptosis *via* deacetylating p53. Our data suggest that high concentration of glucose can induce neuronal apoptosis through downregulation of SIRT1 and increased acetylation of p53, which likely contribute to the development of cognitive impairment in diabetes.

## Introduction

Diabetes has been characterized as a group of metabolic diseases caused by chronic hyperglycemia. Numerous epidemic studies have demonstrated that diabetes increases the risk of the development of cognitive impairment (1–3). With the fast growing number of diabetes and the ever-increasing aging people, diabetic-related diabetic cognitive impairment will challenge public health implication in the future. Therefore, a better insight into the molecular mechanism of the association between diabetes and dementia is of great importance for preventing and slowing the progression of cognitive dysfunction in people with diabetes. Interestingly, several previous studies have indicated that apoptosis in hippocampal neuron promoted the progression of diabetic cognitive dysfunction (4–7). However, the mechanism of neuronal apoptosis in diabetes is still unclear.

Sirtuin 1 (SIRT1), commonly known as a protein deacetylase depended on nicotinamide adenine dinucleotide (NAD^+^), takes part in cell aging (8), differentiation (9), and apoptosis (10). Due to its deacetylating function, some data have indicated that SIRT1 may reduce cell apoptosis through deacetylating p53 (11, 12). p53 responds to various stress signals by modulating the expression of target genes which take part in DNA repair, metabolic pathways, and apoptosis (13, 14). Hyperacetylation of p53 has been shown to be positively associated with pro-apoptotic factors and can be reduced by the deacetylation activity of SIRT1 (15).

Thus, we hypothesized that SIRT1 may get involve in the neuronal apoptosis caused by hyperglycemia in diabetes *via* p53 signal pathway. To confirm the hypothesis, the expression of SIRT1 in streptozotocin (STZ)-induced diabetic mice and high glucose-treated neuroblastoma cells were measured. Then, we investigated whether activation of SIRT1 had a neuroprotective effect against high glucose-induced neuronal apoptosis *via* p53 signal pathway. These findings will offer new insights for diabetic cognitive impairment development and progression.

## Materials and Methods

### Animals and Animal Experiment Procedure

After 1 week of acclimation, 17 C57BL/6 mice (Department of Laboratory Animals, Central South University) were randomly divided into two groups: the control group (*n* = 8) and the diabetes group (*n* = 9). The mice were raised in animal quarters with comfortable temperature and humidity. All procedures involving animal and their care were conducted in conformity with NIH Guidelines for Care and Use of Laboratory Animals. And the experiment was approved by the institutional animal care committee of Central South University.

After overnight fasting, the mice were modeled diabetes by a single intraperitoneal dose of STZ (Sigma, USA). At the same time, an equivalent volume of vehicle was infused into the control group. 0.1 M sodium citrate buffer (pH 4.4) was used to dissolve STZ. According to the previous study, the dose of 220 mg/kg was administered (16). To avoid hypoglycemic shock, 5% glucose was added into the water for 24 h after STZ injection. Three days after the administration of STZ, the blood from the tail vein was collected to measure the blood glucose for all mice. Mice with non-fasting blood glucose ≥16.7 mM in two consecutive tests were considered diabetic.

At the time of sacrifice, mice were anesthetized with pentobarbitol sodium. Then, we exposed the four mice’ skulls from each group along the midline and placed the brain on a precooled cutting board for western blotting and SIRT1 activity test, and dissected hippocampus were snapped frozen in liquid nitrogen and preserved at −70°C. The remaining brains of mice were perfused with 4% paraformaldehyde and were then fixed in same solution for hematoxylin–eosin staining and immunohistochemistry staining.

### Immunohistochemistry

Procedures were conducted according to instruction of the Vectastain ABC elite kit (ZSGB-BIO, China). Briefly, paraformaldehyde-fixed tissue sections were deparaffinized and rehydrated. Then, 10 mM sodium citrate (pH 6.0) was used to expose the antigen. To inactivate endogenous peroxidase, the slides were incubated with 0.3% hydrogen peroxide. Primary antibody was incubated overnight at 4°C after non-specific binding elimination. Then, the samples were treated with biotinylated secondary antibody for 60 min at room temperature, with streptavidin–biotin-horseradish peroxidase for another 60 min. 3,3′-Diaminobenzidine was used to visualize. Finally, the sections were counterstained with hematoxylin.

### Cell Culture and Differentiation

SH-SY5Y cells obtained from the State Key Laboratory of Medical Genetics were used in the experiment. SH-SY5Y cells were cultured in DMEM/F12 (Gibco, USA), 10% fetal bovine serum (FBS; Gibco, USA), and 1% streptopenicillin (Gibco, USA) at 37°C in a humidified cell culture incubator with 5% carbon dioxide. The human neuroblastoma cells were incubated in the medium containing 1% FBS for 24 h and then treated with 10 µM all-*trans*-retinoic acid (RA; Sigma, USA) for 5 days before experiments.

### Apoptosis Assay

According to the result of our previous study, SH-SY5Y cell was cultured in 75 mm glucose for 96 h to mimic the high glucose condition *in vitro*. Cell apoptosis was detected with DAPI nuclear staining. Briefly, after treatment, cells were gently washed with PBS and then stained using DAPI solution (Beyotime, China) for 5 min. The cells were subsequently visualized under a fluorescence microscope (Leica, Germany). To quantify apoptosis, two fields from five wells were blindly analyzed by an observer blinded to the photographers.

### SIRT1 Agonist Treatment

Cells were cultured in 75 mM glucose for 96 h with or without SIRT1 agonist resveratrol (RV) (20 µM; Sigma, USA).

### Western Blot

Proteins were extracted from the whole hippocampus of the mice or SH-SY5Y cells. The protein mass was detected by the bicinchoninic acid protein assay method (Beyotime, China). 50 µg of boiled proteins was separated by SDS-PAGE and blotted with anti-caspase-3 (cleaved form; Abacm, USA), anti-Bax antibody (Abcam, USA), anti-Bcl-2 antibody (Abcam, USA), anti-SIRT1 antibody (Abcam, USA), anti-p53 antibody (Abcam, USA), anti-Ace (K382)-p53 (Abcam, USA), and anti-β tubulin antibody (Abcam, USA).

### SIRT1 Activity

Sirtuin 1 activity was measured by the SIRT1 colorimetric assay kit (Genmed, China) according to the instruction. Simply, synthesized substrate [Arg–His–Lys–Lys (Ac)] was incubated with hippocampus or SH-SY5Y cells. When SIRT1 is active, the acetylated substrate will be deacetylated, resulting in the change of colorimetric absorbance. The absorbance was detected by a M2 plate reader (Tecan, Austria).

### Statistical Analysis

Independent *t*-test or Cochran and Cox *t*-test for continuous data was performed for comparison of two groups. Statistical analyses were performed with SPSS for windows (version 17.0). The significance level α is 0.05.

## Results

### Hippocampus Neuronal Apoptosis in STZ-Induced Diabetic Mice

**Figure 1:**
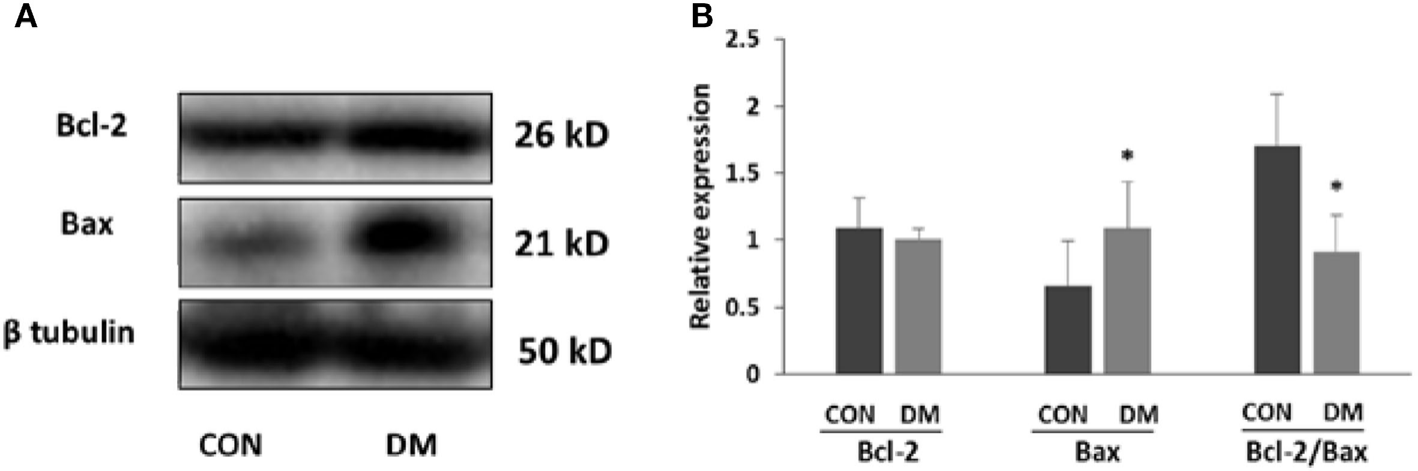
Expression of apoptosis-related proteins in hippocampus of STZ-induced diabetic mice. **(A)** Representative Western blot gel images of Bcl-2 and Bax. **(B)** Summarized data of apoptosis-associated protein Bcl-2 and Bax in the hippocampus of STZ-induced diabetic mice and control. All values are expressed as the mean ± SD. Abbreviations: CON, control; DM, diabetes mellitus. *P < 0.05 versus control. *N* = 4.

At the fourth week after STZ administration, changes in apoptosis-related proteins in hippocampus were examined. The protein levels of Bax (a protein facilitating apoptotic cell death) and Bcl-2 (a protein that is protective of apoptotic cell death) were measured by western blotting. In 4-week diabetic mice, the Bax expression was significantly increased. However, the level of Bcl-2 was not changed in diabetic mice. Nevertheless, our results showed that the ratio of Bcl-2 to Bax was significantly decreased (Figure 1, *P* < 0.05), indicating the enhanced neuronal apoptosis in hyperglycemia mice.

### SIRT1 Is Decreased in Hippocampus Neuron of STZ-Induced Diabetic Mice

**Figure 2:**
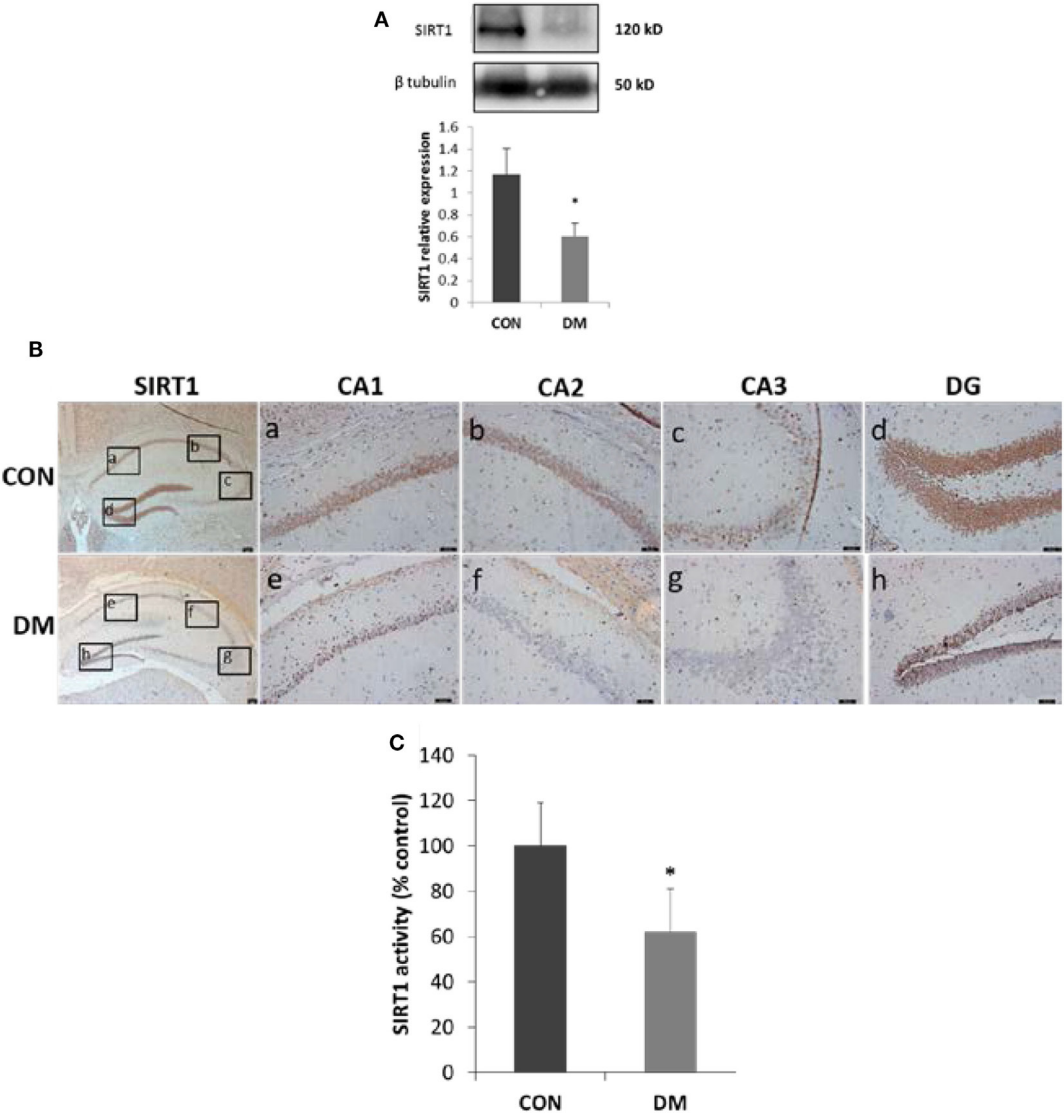
Expression of sirtuin 1 (SIRT1) in hippocampus of STZ-induced diabetic mice. **(A)** Representative Western blot gel images and quantification data of SIRT1 (*N* = 4). **(B)** Immunohistochemical stainings of SIRT1. Represent the stainings of CA1, CA2, and CA3 sectors of the hippocampus and dentate gyrus (DG) in control group or diabetes group is shown in panel [**(B)**, a–d or e–h], respectively. Scale bars: 50 µm. **(C)** SIRT1 activity (*N* = 4). All values are expressed as the mean ± SD. Abbreviations: CON, control; DM, diabetes mellitus. *P < 0.05 versus control.

Sirtuin 1 expression and activity in hippocampus was detected after STZ administration of male C57BL/6J mice. Four weeks after STZ treatment, both SIRT1 levels and activities were decreased in hippocampus. To locate the alteration of SIRT1 in hippocampus, immunostaining was used. We found SIRT1 mainly decreased in cognition-associated areas such as CA1, CA3, and dentate gyrus (DG) (Figure 2).

### Effect of High Glucose on SH-SY5Y Cells Apoptosis

**Figure 3:**
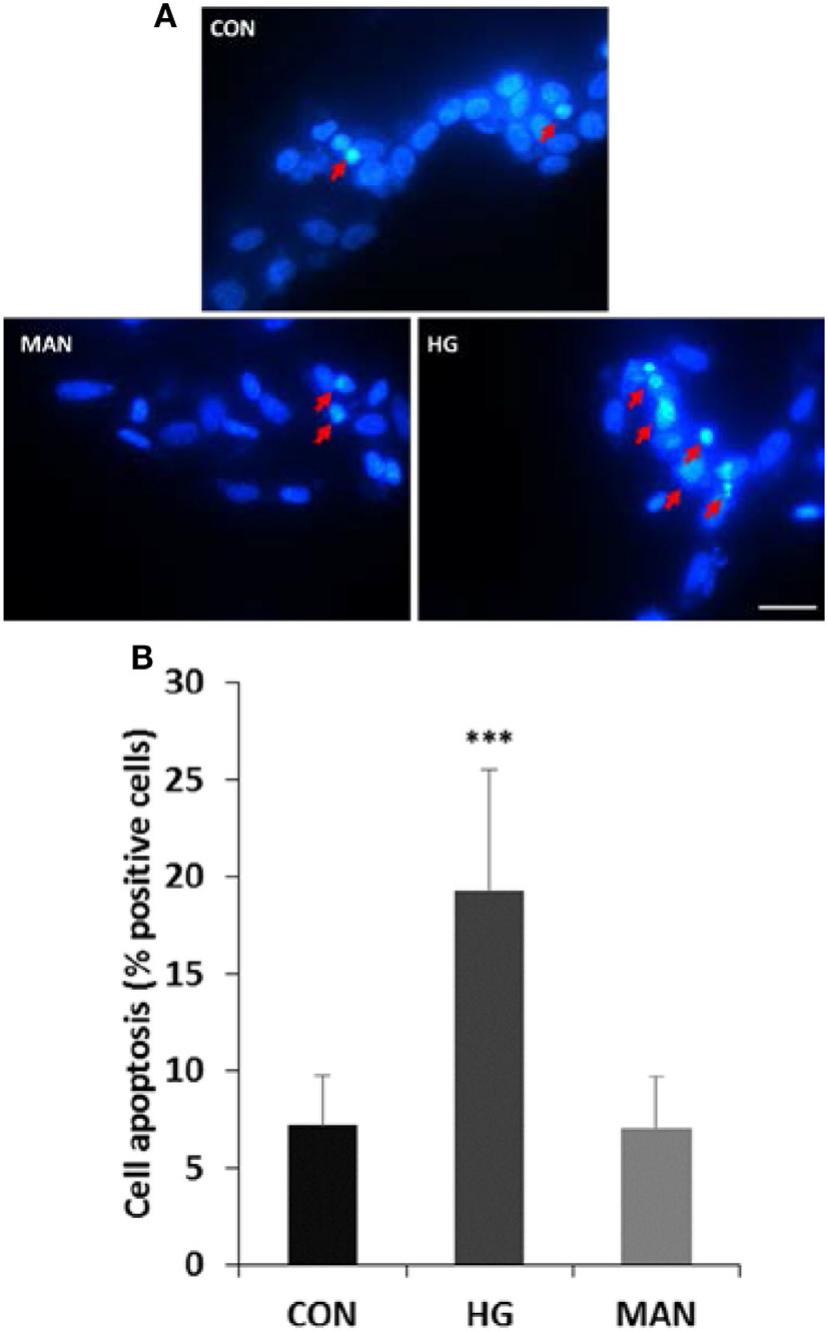
Hyperglycemia induces apoptosis in cultured SH-SY5Y cells. **(A)** Representative fluorescence micrographs of DAPI staining of SH-SY5Y cells exposed to 5.5 mM (CON), 75 mM glucose (HG), or mannitol (MAN) for 96 h. The representative apoptotic cells were marked by arrows. Scale bar: 20 µm. **(B)** Percentage quantification of apoptotic cells. ***P<0.001 versus control.

Differentiated SH-SY5Y cells were cultured in 75 mM glucose or 69.5 mM mannitol (MAN) (plus 5.5 mM glucose in DMEM/F12 medium) for 96 h, and the cell apoptosis was measured with DAPI staining. A small number of apoptotic cells (7.2%) were measured in the control group. However, 19.3% apoptotic cells were detected by DAPI staining after high glucose treatment. Similar with the control group, the apoptotic rate in cells treated with MAN was 7.1% (Figure 3).

### Alteration of SIRT1 and p53 in SH-SY5Y Cells Under High Glucose Condition

**Figure 4:**
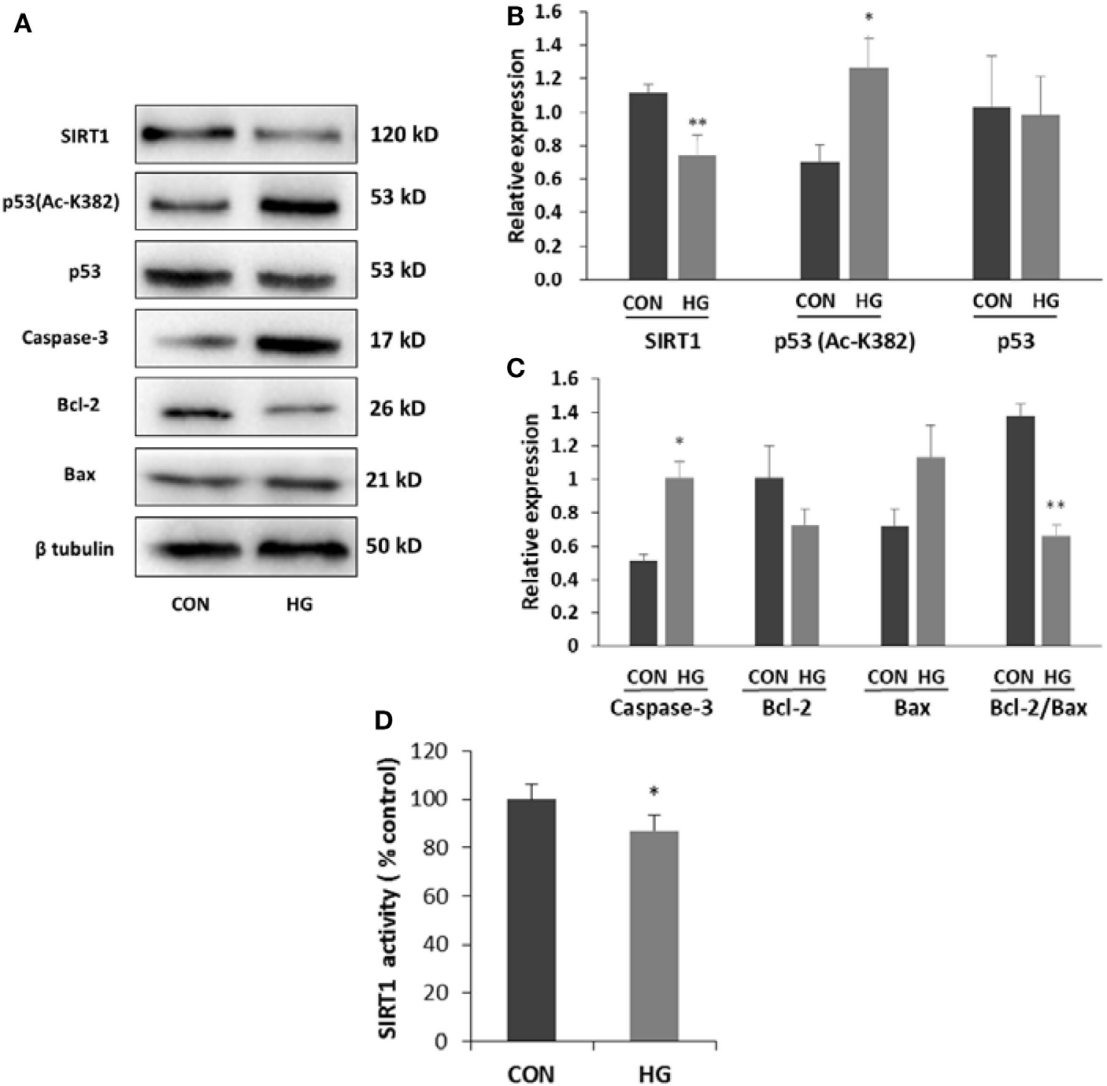
Effects of high glucose on the expressions of sirtuin 1 (SIRT1), p53, Ac-p53, and apoptosis-associated proteins. SH-SY5Y cells were cultured in either 5.5 mM (CON) or 75 mM (HG) glucose-containing media for 96 h. **(A)** Representative Western blot images. **(B)** Quantifications of SIRT1, p53 (Ac-K382), and p53 expression. **(C)** Quantifications of caspase-3, Bcl-2, Bax, and Bcl-2/Bax expression. **(D)** SIRT1 activity. *P < 0.05; **P < 0.01 versus control. *N* = 3.

Differentiated neuroblastoma cells were cultured with 75 mM glucose for 96 h to explore whether high glucose could induce downregulation of SIRT1. Similar with the results in diabetic mice, both activity and expression of SIRT1 decreased significantly in high glucose-incubated SH-SY5Y cells. To investigate the possible mechanism between the downregulation of SIRT1 and cell apoptosis, we examined p53 protein by the Western blotting. The results showed an increase in acetylated p53 protein in cells treated with high glucose (Figure 4). To further characterize the trait of apoptotic mechanism, the status of Bax, Bcl-2, and caspase-3 was tested. SH-SY5Y cells showed a dramatic decrease in the ratio of Bcl-2 to Bax and an increase in the expression of caspase-3 under high glucose condition (Figure 4), suggesting that high glucose-induced apoptosis possibly be mediated by the mitochondrial pathway. Based on the above results, we guessed that SIRT1 may induce neuronal apoptosis through p53/Bax pathway.

### Activation of SIRT1 Alleviated Cell Apoptosis *via* Decreasing p53 Acetylation

**Figure 5:**
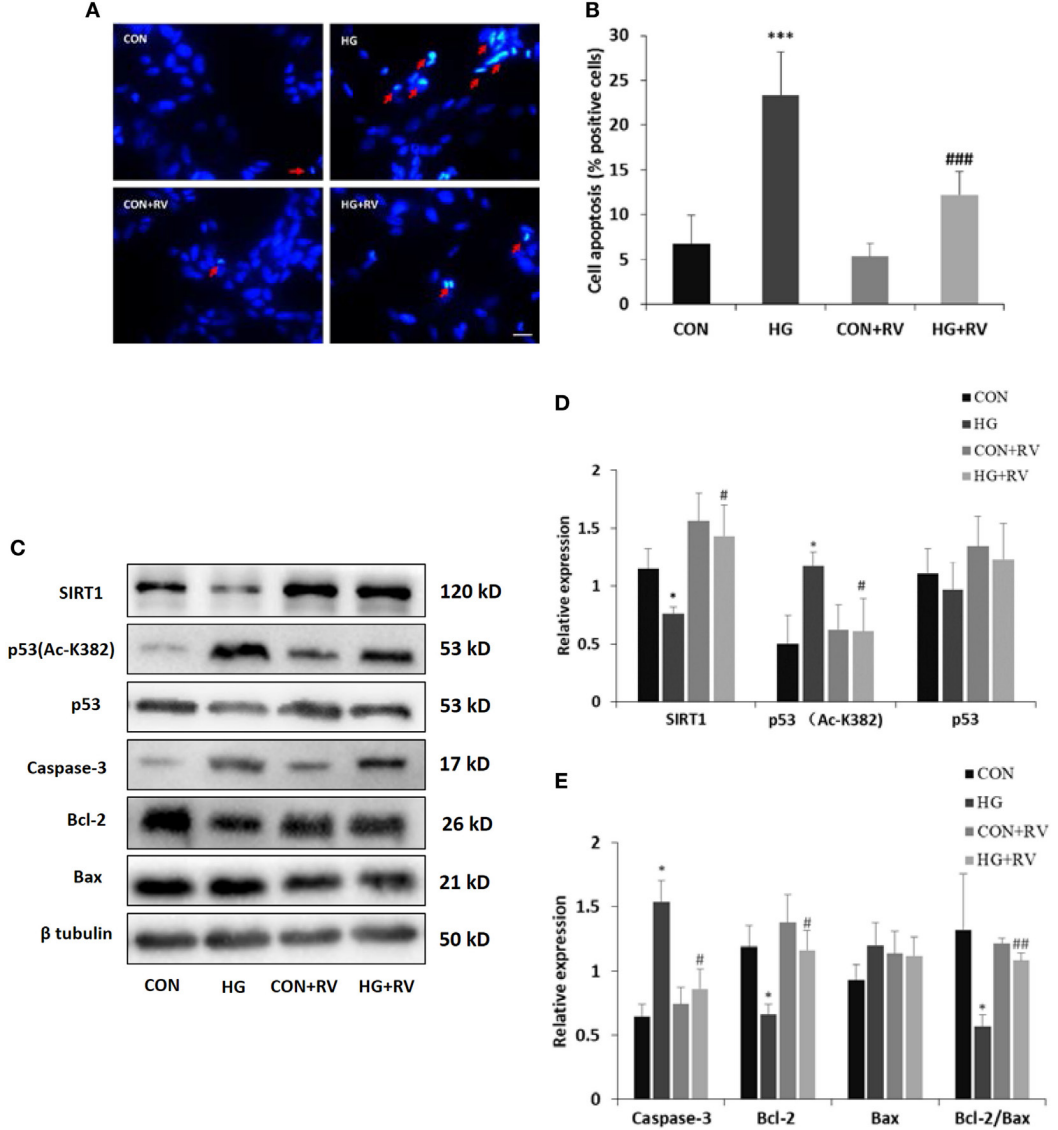
Activation of sirtuin 1 (SIRT1) attenuates high glucose-induced cell apoptosis *via* deacetylating p53. SH-SY5Y cells were cultured in HG media for 96 h in the presence of 10 µM resveratrol (RV). **(A)** Representative fluorescence micrographs of DAPI staining of SH-SY5Y cells treated with or without RV. Arrow denotes cell apoptosis. **(B)** Quantification of cell apoptosis. **(C)** Representative Western blot images of protein expression. **(D)** Quantifications of SIRT1, p53 (Ac-K382), and p53 expression. **(E)** Quantifications of Caspase-3, Bcl-2, Bax, and Bcl-2/Bax expression. *P < 0.05; **P < 0.01; and ***P < 0.001 versus control. ^#^*P* < 0.05; ^##^*P* < 0.01; and ^###^*P* < 0.01 versus HG. Scale bar: 20 µm. *N* = 3.

To explore the effects of SIRT1 activation on the differentiated SH-SY5Y cells, SIRT1 agonist RV was applied. Activation of SIRT1 significantly reduced the amount of apoptotic or necrotic cells induced by high glucose. As shown in Figure 5, the number of cells with positive DAPI staining was 12.3% in the presence of RV. However, without activator, the number reached up to 23.4%.

Then, we examined whether activation of SIRT1 could decrease the acetylated p53 level. Just as results shown in Figure 5, treatment with RV raised the SIRT1 expression and reduced the acetylated p53 level compared with cells incubated with high concentration of glucose.

To further support these findings, several proteins associated with apoptotic cascade were assayed. The high glucose-induced increase of caspase-3 and the decline of the Bcl-2/Bax ratio were both partially reversed in the cells in the presence of SIRT1 activator.

## Discussion

The major aim of our work was to explore whether SIRT1 is involved in neuronal apoptosis induced by diabetes. Using STZ-induced diabetic mice and *in vitro* cultured differentiated SH-SY5Y cells, we found SIRT1 was obviously decreased. Activation of SIRT1 could alleviate high glucose-induced cell apoptosis by deacetylate p53. These findings revealed a novel molecular mechanism involving neuronal apoptosis in diabetes mellitus.

There has been a wide recognition that type 1 DM (T1DM) can cause cognitive impairment (17, 18). STZ is a commonly used reagent to model T1DM due to its preferentially toxic to beta cells in pancreas. Substantial evidence and our previous studies have demonstrated that single high dose of STZ can induced diabetic cognitive dysfunction at early stages of DM (19, 20). Hippocampus, an internal structure of the brain, plays a very important role in several key fields of cognitive function, such as spatial navigation, memory, and learning (21, 22). And previous studies have indicated that hippocampal neuronal apoptosis has close connection with diabetic cognitive impairment (23–26). Therefore, clarify of the mechanism in hippocampal neuronal apoptosis in diabetes will contribute a lot to the prevention and treatment for the diabetic cognitive impairment.

Sirtuin 1 is widely expressed and has been shown to be highly expressed in the developing mouse central nervous system (27), adult mouse and human brain (28). In the adult brain, SIRT1 is expressed in the most brain areas and is prominent in the hippocampal neuron (29), which is replicated in our results. Furthermore, SIRT1 was found highly decreased in the hippocampus with neuronal apoptosis, especially in the CA1, CA3, and DG subregions, which is closely related with memory and learning (30). And the activity of SIRT1 was also decreased. Bcl-2 family consists of a group of cytoplasmic proteins and is notable for its regulation of cell apoptosis. There are two main proteins in this family, Bcl-2 and Bax. However, they are functionally opposite: Bax promotes apoptosis, whereas Bcl-2 counteracts this effect (31). And the ratio of Bcl-2 to Bax controls the cell life after an apoptotic stimulus. We found in this study, the ratio of Bcl-2 to Bax decreased in hippocampus of diabetic mice than that of the controls. These results indicated that hippocampal neuronal apoptosis in STZ-induced diabetic mice appeared to correlate with SIRT1. Thus, further study was conducted *in vitro*.

SH-SY5Y cell line, derived from SK-N-SH, is a widely used model for studying the neuronal mechanism. SH-SY5Y cells are able to differentiate into neuronal lineage by all-*trans-*RA treatment (32). Our previous study have proved that culturing differentiated SH-SY5Y cell in 75 mM glucose for 96 h is an appropriate choice to mimic high glucose condition *in vitro*. Another study also proved that SH-SY5Y cell cultured in high glucose (above 50 mM) occured hyperglycemic stress and cell injury (33). Our results indicated that the apoptotic cells were increased in the high glucose-cultured group. Besides, the ratio of Bcl-2 to Bax and the expression of caspase-3 were both changed in high glucose-cultured neuroblastoma cells. Similar with the findings *in vivo*, these results further indicated that glucose at high concentration could induce cell apoptosis through the mitochondria pathway.

Previous studies have proved that p53 can be deacetylated by SIRT1 by overexpression and dominant-negative strategies both *in vitro* and *in vivo* (11, 15). Multiple lysine residues of p53 can be acetylated in human, including K320, K373, and K382 (11). Acetylation at different residues induces diverse aspects of p53-mediated stress response. Acetylation of p53 on K382 residue facilitates p53 activation, which triggers the transcription of target genes associated with apoptosis (34). Our results showed that the increasing of acetylation of p53 K382 accompanied by decreasing of SIRT1. These findings strongly suggested that the decline of SIRT1 induced by high glucose resulted in acetylation of p53 at K382 lysine residues, which could mediate neuronal apoptosis.

Resveratrol is a well-known agonist of SIRT1 (35). So, we used RV in SH-SY5Y cells with neuron-like phenotypes to further prove the above view. This work indicated that the high glucose-induced decreasing of SIRT1 was reversed with RV. Then, we detected the effect of activation of SIRT1 on cell apoptosis and apoptosis-related protein. The most important finding of this work is that activation of SIRT1 can partially reverse the SH-SY5Y cell apoptosis mediated by high concentration of glucose. The reverse of the apoptosis-related protein, caspase-3 and the ratio of Bcl-2 to Bax, further confirmed it. Some previous studies also reported that RV had protective effect in the neuronal survival (36, 37). Although previous studies indicate that the activation of SIRT1 is a key aspect of RV action, the interaction between RV and other factors cannot be rule out.

Taken together, we demonstrate that decreasing of SIRT1 and neuronal apoptosis occures in the STZ-induced diabetic mice. In high glucose-incubated SH-SY5Y cells, decreasing of SIRT1, p53 acetylation, and cell apoptosis are confirmed, and activation of SIRT1 by RV attenuate the cell apoptosis *via* deacetylation of p53. It is therefore suggested that SIRT1 may be a therapeutic target for the treatment of diabetic cognitive impairment.

## Ethics Statement

All procedures involving animal and their care were conducted in conformity with NIH Guidelines for Care and Use of Laboratory Animals, and the experiment was approved by the institutional animal care committee of Central South University.

## Author Contributions

JW and SW designed the experiments. The experimental procedures were performed by XS, LP, SZ, XL, and FM. XS and JW prepared the manuscript. ZL revised the manuscript.

## Conflict of Interest Statement

The authors declare that the research was conducted in the absence of any commercial or financial relationships that could be construed as a potential conflict of interest.
